# Annual Cycle and Migration Strategies of a Trans-Saharan Migratory Songbird: A Geolocator Study in the Great Reed Warbler

**DOI:** 10.1371/journal.pone.0079209

**Published:** 2013-10-18

**Authors:** Hilger W. Lemke, Maja Tarka, Raymond H. G. Klaassen, Mikael Åkesson, Staffan Bensch, Dennis Hasselquist, Bengt Hansson

**Affiliations:** 1 Molecular Ecology and Evolution Laboratory, Department of Biology, Lund University, Lund, Sweden; 2 Montagu’s Harrier Foundation and Animal Ecology Group, Centre for Ecological and Evolutionary Studies, Groningen University, Groningen, Netherlands; 3 Grimsö Wildlife Research Station, Department of Ecology, Swedish University of Agricultural Sciences, Riddarhyttan, Sweden; University of Milan, Italy

## Abstract

Recent technological advancements now allow us to obtain geographical position data for a wide range of animal movements. Here we used light-level geolocators to study the annual migration cycle in great reed warblers (*Acrocephalus arundinaceus*), a passerine bird breeding in Eurasia and wintering in sub-Saharan Africa. We were specifically interested in seasonal strategies in routes and schedules of migration. We found that the great reed warblers (all males, no females were included) migrated from the Swedish breeding site in early August. After spending up to three weeks at scattered stopover sites in central to south-eastern Europe, they resumed migration and crossed the Mediterranean Sea and Sahara Desert without lengthy stopovers. They then spread out over a large overwintering area and each bird utilised two (or even three) main wintering sites that were spatially separated by a distinct mid-winter movement. Spring migration initiation date differed widely between individuals (1-27 April). Several males took a more westerly route over the Sahara in spring than in autumn, and in general there were fewer long-distance travels and more frequent shorter stopovers, including one in northern Africa, in spring. The shorter stopovers made spring migration on average faster than autumn migration. There was a strong correlation between the spring departure dates from wintering sites and the arrival dates at the breeding ground. All males had a high migration speed in spring despite large variation in departure dates, indicating a time-minimization strategy to achieve an early arrival at the breeding site; the latter being decisive for high reproductive success in great reed warblers. Our results have important implications for the understanding of long-distance migrants’ ability to predict conditions at distant breeding sites and adapt to rapid environmental change.

## Introduction

 Many species of birds, turtles and sea mammals conduct astonishing long-distance journeys between their breeding and wintering grounds [1]. These impressive migration events have fascinated man for centuries and modern tracking techniques such as GPS-loggers and light-level geolocators are now revealing details on route characteristics and migratory behaviours that are revolutionizing our understanding of fundamental aspects of migration [2], [3].

 The timing of annual events is of prime importance for migratory species. For example, arriving early at the breeding site allows settling in superior territories, thereby improving mating and reproductive success [4–6]. However, early migration may also entice costs in terms of delays and higher risk of mortality (e.g. [7]). Thus, choosing optimal wintering areas, migration routes and strategies have important consequences for survival and fitness [3], [8]. It is interesting to note that spring migration to the breeding grounds generally seem to proceed faster than the autumn migration to the wintering grounds [8], a pattern that has recently been supported by geolocator studies in several birds including northern wheatear (*Oenante oenante*), red-backed shrike (*Lanius collurio*) and common swift (*Apus apus*) [9–11]. Faster travel speed in spring may reflect the importance of early arrival to the breeding sites. Recent geolocator studies have also demonstrated a high degree of individual variation in how migration is organized in terms of route and timing [12–15]. For example, hoopoes (*Upupa epops*) from a single breeding population in Switzerland chose widely scattered wintering sites in western and eastern Sahel, respectively [14], and golden-crowned sparrows (*Zonotrichia atricapilla*) wintering on the central coast of California (USA) settled at different localities along the coast of the Gulf of Alaska [16] (see also 17], [18). Moreover, Swainson’s thrushes (*Catharus ustulatus*) from a narrow breeding area in south-western Canada exhibited longitudinally separated migratory routes on their southward migration over the North American continent [19] (see also 20). Quantifying general and individual seasonal strategies in routes and schedules of migration are important to understand how events in the annual cycle interact and how events during one stage can ‘carry-over’ into subsequent stages, such as reproduction [21], [22]. An interesting example of how harsh environmental conditions during migration can cause substantial delays in breeding was the severe drought in the Horn of Africa in 2011 that affected the timing of the spring migration of several long-distance migrants including geolocator-equipped red-backed shrikes and thrush nightingales (*Luscinia luscinia*) [7].

The great reed warbler (*Acrocephalus arundinaceus*) is a long-distance migrant that breeds in reed marshes in Europe and western Asia and winters over the whole of sub-Saharan Africa [23–25]. In the present study, we used geolocators to study migrations of male great reed warblers from a breeding area at the northern edge of the species’ breeding range in southern Central Sweden where we have performed a long-term study of the species’ breeding ecology since the mid-1980s (e.g. [26–29]). Ringing recoveries from the population indicate a southerly directed migration route via the Central Mediterranean region and a wintering area ranging at least between the Ivory Coast in the west to southern Chad in the east [30]. These occasional recoveries represent the only available data to infer the migratory pattern of Swedish great reed warblers. Here, we were particularly interested in using tracking data to evaluate (i) whether there exist prominent differences between autumn and spring migration with respect to routes, speed and timing, (ii) to what extent individuals may utilise multiple wintering sites rather than single sites or even have a nomadic life-style, and (iii) the size of the African wintering range of these males breeding in Sweden. These geolocator data can fill an essential gap in our knowledge of this well-studied songbird population, where detailed and long-term breeding performance and fitness data are available [26–28]. Moreover, answers to these questions have broad implications in the context of evolution of migratory strategies, adaptation to environmental changes and conservation of migratory species [3], [31], [32]. 

## Methods

We have studied great reed warblers at Lake Kvismaren in southern Central Sweden (59°10’N, 15°24’E) on a daily basis throughout the breeding seasons since 1983 [26], [28], [33]. In this population nearly all of the breeding birds (approximately 50 individuals annually) have been captured and marked with an aluminium ring and individual-specific colour ring combinations. Similar studies have also been conducted at nearby Segersjö Bay, c. 15 km from Lake Kvismaren, since 1987, albeit not on a daily basis [34]. In the present study these localities are collectively referred to as the breeding site. Both males and females are highly philopatric to the breeding site and the majority of adults that survive the winter return to breed in the same area [27,34]. The field work at Lake Kvismaren (which has been described in detail elsewhere [26–29]) and the present light-logger study on this non-endangered species have been conducted according to national and international guidelines and have been approved by the Kvismare Bird Observatory, the County Administrative Board and the Lund/Malmö Animal Review Board. 

In 2008 and 2009, male great reed warblers that were known to be successful breeders from at least the preceding season were fitted with a geolocator (2008: n = 9; 2009: n = 26). We used geolocator model Mk10S in 2008 and Mk12-SAD in 2009 (British Antarctic Survey (BAS), Cambridge, UK; www.birdtracker.co.uk). The light sensor was fitted on an 8 mm stalk pointing up and backwards (30° angle) in order to minimise shading effects caused by the bird’s plumage. The geolocators measured light intensity every minute, but stored only the maximum value for each 10 minute interval. The geolocator was mounted on the bird’s back using leg-loop harnesses [35]. The lengths of the plastic strings were fitted individually in the field and cyanoacrylate adhesive were added to seal the knot. The geolocator plus attaching material weighed approximately 1.0 g Mk12-SAD and 1.3 g Mk10S which is less than 4% of the lean body mass (fat score 0; [36]) of male great reed warblers from this study site (in 2010: mean±SD = 33.9±2.4 g, n = 7). 

We used *BASTrak* (BAS) for downloading data from retrieved geolocators and to correct for clock drift (which affected all geolocators over the whole period of data logging). After decompressing the raw data, *TransEdit2* (BAS) was used to visualise light data. A light threshold value of 2 was chosen at which the program assigned transitions between light and dark phases (sunrises and sunsets). To establish the sun azimuth angle corresponding to this threshold value, we used three different methods. First, we used *LocatorAid* (BAS) to calculate the sun angle for the periods when the birds were at a known position (i.e. breeding site) for the year when the geolocator got attached (from date of affixing the geolocator in May to 5 July), and one year later when the birds returned to the study site (from arrival date until date of retrieving the geolocator). Secondly, we used the Hill–Ekstrom calibration procedure, which is based on the observation that the latitude error of the determined fixes increases with increasing mismatch between light threshold value and inferred sun angle. The latitude error is of opposing magnitude on either side of an equinox, so it is possible to evaluate the sun angle by assessing the effect of different sun angles on the latitude curve and choosing the one that minimises these distortions [10], [37]. The Hill–Ekstrom calibration was used during the time of equinoxes when the birds were stationary (inferred from longitude data), i.e. during the long wintering stays in Africa. Finally, to achieve the best possible estimation of the sun angle during the autumn and spring migration the following steps were undertaken. The stationary positions for which the sun angle calibration was determined based either on known position (i.e. the breeding ground) and the Hill–Ekstrom method (i.e. wintering grounds) were plotted in *ArcMap* (ESRI). Migration tracks between breeding and non-breeding grounds were then plotted using different sun angles (0.5 degree intervals within the range of sun angles for the stationary positions) to determine which sun angle provided the best fitting track which minimises the (geographic) mismatch of migration track and the known (stationary) positions of the departure and arrival sites. The sun angle calibration technique was used for autumn and spring migration tracks individually for all birds. Sun angles used for each individual are given in Table S1. For each file, time of sunset was advanced by 9 min to correct for the time lag due to the logging interval. *Locator* (BAS) was used to calculate two ‘fix positions’ (referred to as fix points or fixes below) per day (noon and midnight) from the sunrise/sunset times. 

In order to determine periods when equinoxes had negative effects on the accuracy of the data, each bird’s longitude and latitude positions were plotted on a graph and visually checked for erratic patterns during times of equinoxes (between 18 and 42 days of data around the equinoxes were excluded). Before plotting the tracks on a map, the original positions were smoothed twice by applying a running average for each fix point and its two adjacent fix points on either side of the focal fix point (thus covering a 36 hour period) [38]. 

We estimated the accuracy of the calculated position data by comparing the distance between the true position of the breeding sites and the inferred geolocator positions for the same periods as used for the sun angle calibration (see above). The estimation of the accuracy for the calculated positions on the breeding grounds resulted in a mean error distance for all individuals of 205.7 km (median = 103.9 km, SD = 296.1 km, *n* = 10; estimates for 6 individuals prior to departure and after arrival and for 4 individuals only before departure; Table S2). These estimates included a few highly erroneous data points clearly visible when inspecting the data in *ArcMap* (*ArcView 9.3* (ESRI)) – namely 9 positions for individual 1V-87 that were larger than 3 times the SD of the distance error for all breeding site positions of all birds (i.e. > 3 × 884.4 km). When these extreme positions were excluded the estimation of the accuracy resulted in a mean error distance for all individuals of 134.2 km (median = 101.1 km, SD = 116.4 km, *n* = 10; Table S2). The accuracy of our data (Table S2) lays well within the range of position error estimates known from other geolocator studies. It is expected that the accuracy of positions calculated with sun angles calibrated with the Hill–Ekstrom method is less precise, since the error for position calculations increases towards the equator. Thus, migratory tracks and stationary positions determined in this study should be regarded as estimates where an error of approximately 250 km is possible (cf. [38–40]). 

Active migratory movements were differentiated from stopovers by their clearly directional pattern compared to the clustered pattern of stopovers. Only a cluster of at least 3 fixes (36 hours), which then was laying within a 210 km radius, i.e. roughly twice the median error distance for precision estimates (without the extreme outliers observed in 1V-87, see above and Table S2), were defined as stopover. When birds were stationary at stopover, moulting and wintering sites, we used the average value of the positions to plot the geographic position. Great-circle distances were calculated cumulatively between smoothed positions (12h segments) for all migratory tracks. In cases where data points were missing during movements (i.e. only during start or end of an equinox), the great-circle distance between available positions flanking a gap was used (see Figure S1). Travel speed (i.e., during active migration) was calculated by dividing the cumulative distance covered with directionally positioned fix points by the number of days. Migration speed was calculated as the total distance (km) covered divided by the total time (days) used, either from the breeding site to the first wintering site (autumn migration), or between the last wintering site and the breeding site (spring migration). Because year-related differences were not possible to detect due to low sample size of the study season 2008/2009 (n = 2) compared to 2009/2010 (n = 8), the data of both study seasons were pooled for all statistical analyses. All statistical tests (t-tests, paired t-tests and Spearman’s rank correlations) are two-tailed. The length of the light phase of a day for the mid-latitude between breeding and determined wintering site, on the mid-date of the migration period, was calculated using an online calculator (http://www.dawnsun.net/astro/suncalc/).

## Results

We recovered and downloaded geolocator data for 10 out of 37 males (2 of 9 males in 2008/2009, and 8 of 26 males in 2009/2010). These between-year return rates were similar to non-logger attached males (2 out of 8 males in 2008/9, and 2 out of 6 males in 2009/10). Due to technical problems with some of the geolocators, complete data over the full annual cycle were available for 6 males (Figure S1a-f), autumn and partial wintering data for 2 males (Figure S1g,i), and parts of the autumn migration for 2 males (Figure S1h,j).

### Spatial patterns

**Figure 1:**
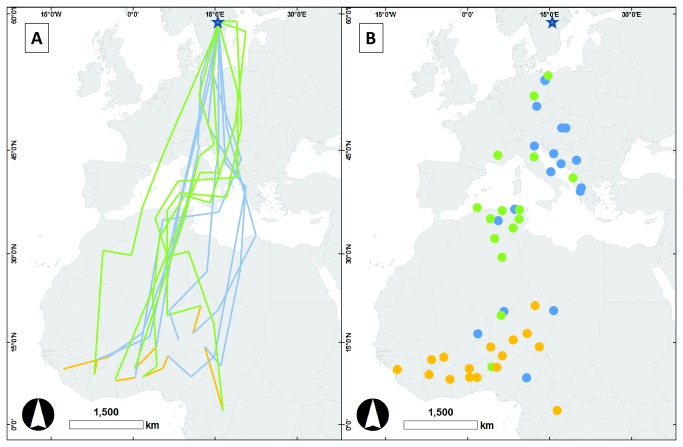
Inferred migration routes, mid-winter movements and stopover sites from geolocator data of male great reed warblers. (A) Migration routes and mid-winter movements (blue: autumn; green: spring; yellow: mid-winter). (B) Stopover sites (stays for more than 36-hours) in autumn (blue) and spring (green), and wintering sites (yellow). Breeding site is indicated (star). Data are for 8 males in autumn and winter, and 6 males in spring. For details of individual birds, see Figure S1.

On autumn migration, the great reed warblers followed a southerly directed route to their first stopover site that showed a wide distribution with stopovers located between north-eastern Germany and north-western Greece (Figure 1a, Figure S1). Four out of ten birds conducted a shorter flight to a second stopover site further south in Europe (H1-25, 7V-89, 1V-87 and 9H-50; Figure 1b, Figure S1e,f,g,i). However, the majority of males crossed the Mediterranean Sea and Sahara Desert without stopovers in a flight that exceeded 24 hours in duration, in a geographical window spanning from Tunisia/Algeria in the west to Libya in the east (Figure 1a). Two birds showed a different pattern: after crossing the Mediterranean Sea, 7V-50 and 9H-50 interrupted their migration for more than 24h in northern Tunisia, before continuing south over the Sahara (Figure 1b, Figure S1c,f).

**Figure 2:**
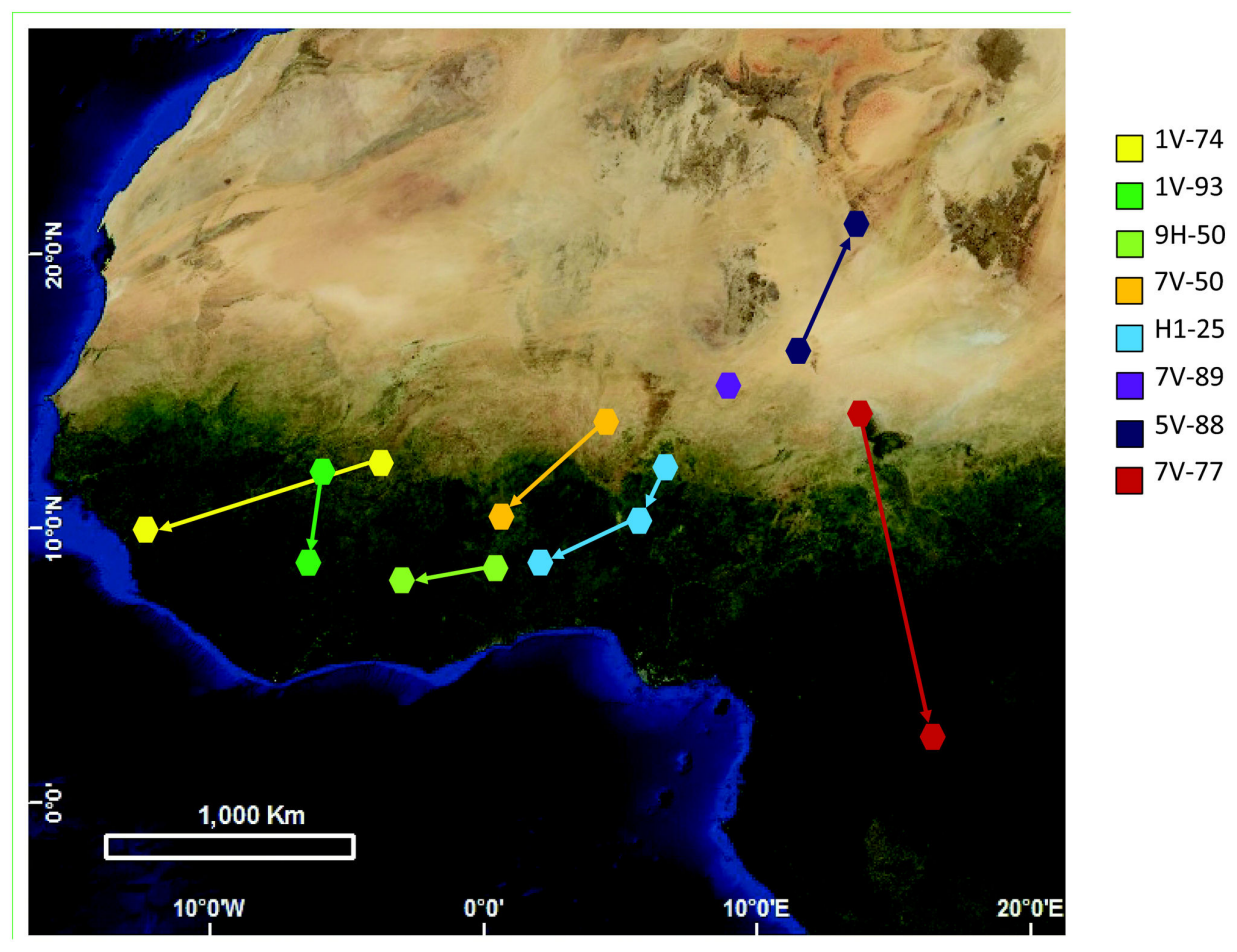
Main overwintering locations and mid-winter movements of individual male great reed warblers. For 7V-89 only the first wintering site could be determined (purple), because the geolocator stopped logging 27 November, i.e., before the movement to the second wintering site. Note that all males with available data did a mid-winter migration to a new wintering site in December - January.

Wintering sites were located throughout the western part of the species’ sub-Saharan wintering range, from West Africa into Central Africa, from Senegal in the west to Chad in the north, and the Congo basin in the east (between 21.1°–2.4°N and -13.0°–16.4°E; Figure 1b). The individuals spent longer periods (several weeks to months) at two (and in one case three) different wintering sites (Figure 2; Figure S1). At the first site (the presumed moulting site) they generally stayed from September to December and at the second site from December to April (Figure S1). The second wintering site was on average located slightly south-west of the first site (Figure 2), but there was no significant difference in mean position between them (first site: mean position 12.8°N, 3.9°E, n = 8; second site: mean position 10.1°N, 1.6°E, n = 7; latitude: t = 1.27, df = 13, p = 0.23; longitude: t = 0.50, df = 13, p = 0.63; Table S3). The mean distance between an individual’s main wintering sites was 678 km (great circle distance; range 351-1352 km, n = 7; Table S3). 

Spring migration routes over the Sahara Desert were generally located more to the west than the southward Sahara crossing in autumn (Figure 1a; clearly so in four males, whereas two males showed similar latitudes in autumn and spring, Figure S1a-f). In spring, after crossing the Sahara desert, all great reed warbler males stopped just south of the Mediterranean Sea in northeast Algeria and western Tunisia (Figure 1b). From the stopover in North Africa most males took off in a north-easterly direction towards Italy and Balkan, which allowed these birds to pass east of the Alps and to return more or less on the same track through Europe as taken in autumn (Figure 1a, Figure S1). Only one out of six males, 1V-93, did not perform an eastern ‘detour’ via Italy and Balkan and instead headed directly north from Algeria crossing the western parts of the Alps taking the shortest straight route towards the breeding site in southern Central Sweden (Figure 1a, Figure S1a).

### Temporal patterns

**Figure 3:**
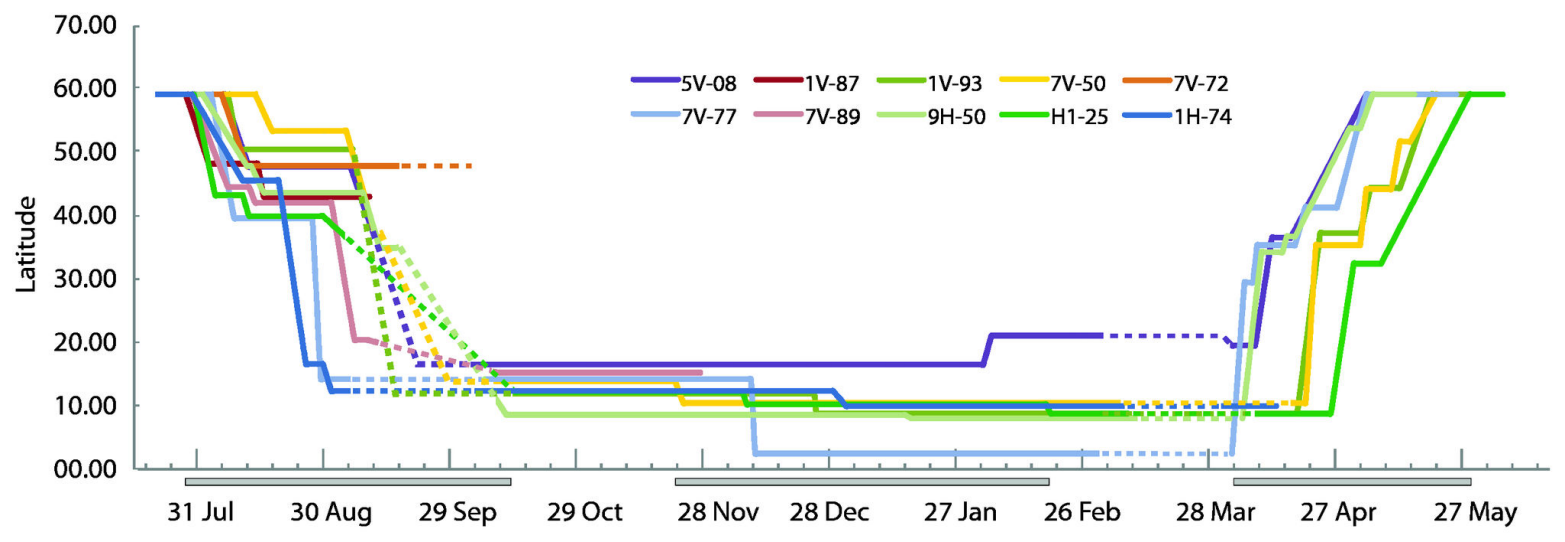
Latitudinal position over time for male great reed warblers, indicating main stopover sites, wintering area and related phenological patterns. Dashed lines indicate uncertainties associated with equinox. The length of each line represents the period and duration of successful working of the geolocator. Grey lines along the x-axis indicate population range for duration of autumn migration, mid-winter movement and spring migration.

The departure date from the breeding grounds ranged between 27 July and 14 August with a mean date of 2 August, whereas the time period from the breeding site to the last stopover site in Europe varied substantially between individuals (Figure 3; Table S3). The mean duration of the last stopover before crossing the Mediterranean Sea and Sahara Desert was 19.5 days (range 9-41 days). The departure date for the southward crossing of the large barriers (the Mediterranean Sea and the Sahara Desert) ranged between 19 August and 7 September with a mean of 31 August. The crossing of the Mediterranean Sea and Sahara Desert to the first stopover site south of the Sahara took 2-7 days (mean = 4.6 days), and the birds arrived at the first wintering site from 29 August to 29 September, with a mean arrival date of 12 September (Table S3). Arrival date at the first wintering site was not significantly correlated with the time of departure from the breeding site (r_S_ = 0.64, n = 8, p = 0.09). Note, however, that there is a tendency for a significant relationship with a correlation coefficient that is relatively high and a statistical power that is rather low due to low sample size.

The timing of the mid-winter movements was unsynchronized with departure dates from the first to the second wintering site from 21 November to 2 February (Figure 3, Table S3). On average, the birds spent equally long periods at each site (101 days at first and 98 days at second wintering site). One bird, H1-25, spent longer periods at three wintering sites (90, 71 and 68 days, respectively; Figure 1b; Figure S1e). The mid-winter movements between the (in most cases) two wintering sites were fast (0.5-2 days travelling 235-1352km), suggesting a relatively fast mid-winter movement. Spring migration was initiated between 1 and 27 April (mean 12 April), and the date of arrival at the breeding ground ranged between 5 and 29 May (mean 14 May; Figure 3; Table S3). There was a strong correlation between the departure date from the last wintering site and the time of arrival at the breeding site (r_S_ = 0.94, n = 6, p = 0.03; Figure 3, Figure S1a-f, Table S3). In contrast, there was no relationship between departure for autumn migration and arrival on the breeding grounds the year after (r_S_ = 0.09, n = 6, p = 0.84).

**Figure 4:**
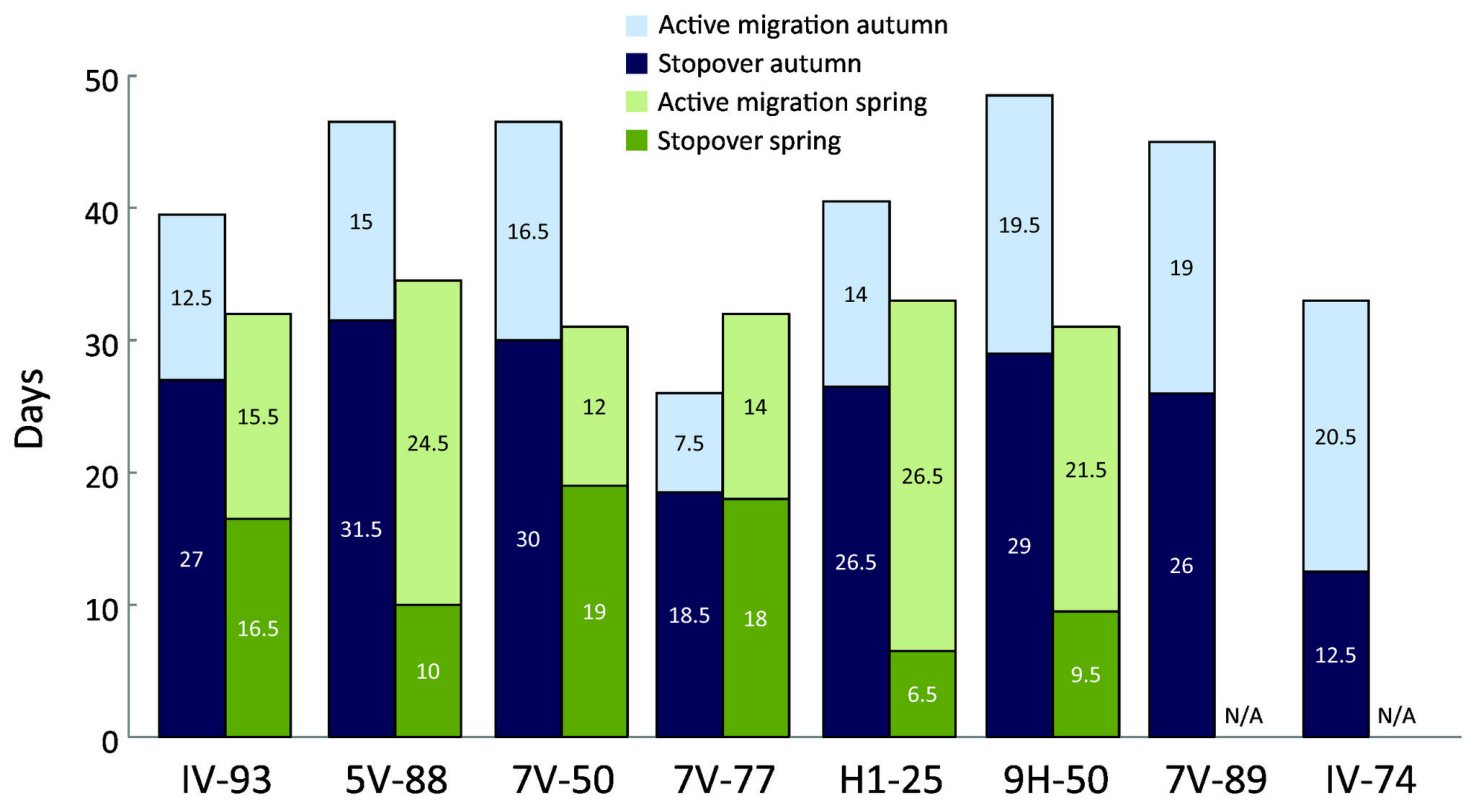
Time allocation of active migration and stopover during autumn (dark and light blue) and spring (dark and light green) for individual great reed warblers.

The speed of migration of individuals for which we had data over the whole route (n = 6) was on average 62% faster in spring (mean 220 km/day) than in autumn (mean 139 km/day; paired t-test: t = 8.68, df = 5, p < 0.001; Table S3). The number of days spent at stopover sites was fewer in spring (mean 13.3 days) than in autumn (mean 27.1 days; paired t-test: t = 4.20, df = 5, p = 0.009), but note a weak tendency for fewer flight days in autumn (mean 14.2 days) than in spring (mean 19.0 days) (paired t-test: t = 1.96, df = 5, p = 0.107; Table S3). These patterns resulted in different migration strategies in spring and autumn where great reed warbler males had a mean travel to stopover ratio of 1:2.0 in autumn as compared to 1:0.7 in spring (Figure 4, Table S3). Overall, in individuals for which we had data over the whole route (n = 6), the average duration of the autumn migration was 41.3 days, which was significantly longer than the spring migration which took on average 32.3 days (paired t-test: t = 2.62, df = 5, p = 0.047; Figure 4, Table S3). The time of arrival at the breeding site did not correlate with duration of spring migration (r_S_ = 0.08, n = 6, p = 0.88).

## Discussion

Our study provides unique data over the annual migratory cycle of trans-Saharan long-distance migranting great reed warblers equipped with geolocators. Our main findings include pronounced differences between autumn and spring migration patters, strong correlation between spring departure and arrival at the breeding site and evidence for two, and in one case three, main wintering sites separated by distinct and fast mid-winter movements, suggesting a true migration journey and not slow continuous movements along the ground searching for suitable habitats. Geolocator position data are associated with possible errors of up to 200-300 km (cf. [38–40]), which implies that small-scale interpretations, such as the exact positions of single stop-over sites, need to be considered with caution. However, the level of data accuracy is sufficient for robust inferences of large-scale interpretations, such as our conclusion about a wide over-wintering range in great reed warblers. Moreover, because the position errors should be similar in autumn and spring there is no reason to expect biases in the comparisons between autumn and spring migration patterns. Unfortunately, four of the 10 geolocators had technical problems and stopped logging before the end of the full year cycle. According to the manufacturer’s instructions such complications may happen due to low ambient temperatures (< -10°C) and/or concussion and vibration [41].

The departure of the birds from the breeding site in southern Central Sweden occurred surprisingly early, already in early August. Feeding conditions at the breeding grounds are favourable at least for another 2-3 weeks in August, which indicates that deteriorated conditions at the breeding site were not the underlying cause for the early timing of autumn migration in male great reed warblers. After leaving the breeding grounds, most individuals chose stopover sites in south-eastern Central Europe, which fits the distribution of earlier ringing recoveries of birds from our study population [30]. When leaving Europe and embarking on the barrier crossings over the Mediterranean Sea and the Sahara Desert the great reed warblers increased their travel speed. Six individuals continued with high speed throughout the crossing of these large barriers until they reached the sub-Saharan zone, whereas two males included a longer stopover (2-5 days) in Tunisia, where ecological conditions might still be favourable during this time of the year. 

Our data clearly show that individual great reed warblers utilize two, or in one case three, main wintering sites, and the presence of a distinct mid-winter movement. The occurrence of a mid-winter movement is in line with De Roo and Deheegher’s [23] observation of a strong influx of great reed warblers at their study site in Zaire (Democratic Republic of Congo) in December, and the observations of considerable fat accumulation, evening migratory restlessness and the disappearance from the area of previously stationary great reed warblers in northern Ghana in December [42]. In Uganda and Ghana, great reed warblers are moulting in October and November [42], [43], which suggests that the first wintering site is used for the complete moult (incl. wing and tail feathers), and that the mid-winter movement takes place on newly moulted flight feathers. The two winter ringing recoveries in Ivory Coast and southern Chad, respectively, of great reed warblers from our Swedish study population suggested a relatively large sub-Saharan wintering area [30]. Nevertheless, our geolocator study revealed that the birds spread out over an even larger wintering range – covering the whole of sub-Saharan West Africa, east to the Congo basin. Such an exceedingly wide west–east wintering range of these birds breeding from a single breeding area in south Central Sweden strongly implies weak migratory connectivity in West European great reed warblers (Figure 2). Wide wintering ranges and a remarkable weak migratory connectivity are suggested also for hoopoes breeding in Europe [14]. 

A completely unexpected result – although an older source had noted an increase of great reed warblers in Tunisia during spring [44] – was that all males spent 1-2 weeks at the end of April or beginning of May in a rather restricted area in north-eastern Algeria and western Tunisia, independent of where along the west–east axis of sub-Saharan Africa they had spent their second part of the winter. These data suggest a general migratory corridor through central West Africa in spring, possibly due to the occurrence of suitable staging during this time of the year (cf. [44]). From the stopover sites in Algeria/Tunisia, some individuals showed a more or less pronounced turn towards east, where the males moved 1,000-1,500 km east–northeast reaching the Balkan region before turning north. This rather long detour towards the east added a considerable extra distance, and hence probably additional energy demands, but could nevertheless be favourable because it would allow circumventing the Alps (cf. [45]) and allow most individuals to re-emerge at their autumn migration track if previous knowledge of stop-over sites and routes would be favourable to them (resulting in a ‘hang-loop’ migration pattern). A combination of favourable wind and feeding conditions has been suggested to underlie the prominent detour across the Arabian Peninsula in the red-backed shrike [10] and over West Africa in the common swift [11]. 

The speed of spring migration in the great reed warblers was relatively constant over long distances and in total faster than during autumn. A similar pattern has also been seen in other bird species [8,11], [46]. That the spring migration is faster and shows less variation in duration between individuals than the autumn migration, suggest that there is increased pressure to arrive early at the breeding grounds and that the achieved migration speed may be close to the maximal possible speed (cf. [47]). The flight to stopover ratio in both autumn and spring (1:2.0 and 1:0.7, respectively) are much lower than the theoretically expected ratio (i.e. approx. 1:7) [48] suggesting that the great reed warblers presumably fuel a lot before their flights when they are still at their breeding and at their wintering grounds, and also feed during travel days particularly in spring. At least three hypotheses may explain the observed increase in migration speed in spring compared to in autumn. First, the ‘day-length hypothesis’ which suggests that the increase in day length during spring migration offers increased foraging opportunities and energy intake for European songbirds [49]. Day length measured at the mean date (28 April) and mean latitude (34.6°N) of all tracked spring migrations (daylight hours 13h 34min) was 19% longer in spring than in autumn (date 23 August, latitude 36.0°N, daylight hours 11h 26min). Secondly, according to the ‘early arrival hypothesis’ high speed of spring migration may reflect strong selection on early arrival for males [4], [50]. This is a highly relevant hypothesis, because early arrival at their breeding site enhances mating success and reproductive output in great reed warblers [6]. Third, the ‘wind hypothesis’ proposes that prevailing wind conditions over central Europe are more favourable during spring than in autumn for north-easterly directed migration of nocturnal migrants [51]. However, the wind conditions are thought to be adverse for the crossing of the Sahara Desert in spring [44]. Clearly, detailed analyses linking meteorological data with track data are needed. 

The departure dates from the wintering site differed substantially between great reed warbler individuals and correlated strongly with the arrival date at the breeding sites (r_S_ = 0.94), and this has also been found in red-backed shrikes, another trans-African long-distance migrant [10]. A pronounced departure–arrival correlation means that late departing individuals do not compensate their delay with an increased migration speed to arrive early, which contrasts the finding in a cross-species study of European *Sylvia* warblers, where late departing species migrated faster to their breeding grounds [52]. The wide span in departure dates that we observed between individuals suggests that departure date is not strongly linked to breeding latitude. However, our data comes from a single population only, so the departure–arrival correlation needs to be followed up with data from additional populations. It is also possible that the individual variation in departure dates from the wintering grounds that we observe may reflect inherent individual differences in their annual schedules (perhaps driven by quality differences) and/or local differences in ecological conditions, prompting some individuals to wait and delay departure until the optimal physiological and meteorological conditions for onset of migration are given [53], [54]. The pronounced departure–arrival correlation and the consistent high speed during spring suggest that the take-off from the African wintering grounds poses an important decision with potential effects at later stages in the annual cycle including timing of breeding and reproductive success. The arrival date differs widely between males and early arrival is of documented importance for successful establishment at the breeding grounds in terms of mating success and reproductive outcome of great reed warblers (e.g. [27]). The obvious uncertainties for birds in foreseeing the conditions at the breeding grounds already at departure in Africa may lead to mistiming between food requirement and food availability, as observed in some trans-African passerines [55], [56]. Future studies linking tracking data with breeding data may improve our understanding of how long-distance migrants may or may not be able to adapt to on-going environmental and phenological changes (cf. [32]). 

To conclude, our findings are consistent with the hypothesis that ecological barriers strongly influence migratory strategies; in the case of the great reed warbler, crossing the Mediterranean Sea and the Sahara Desert was clearly associated with increased travel speed and brief stopover periods. We further conclude (i) that there exist prominent differences between autumn and spring migration with respect to stopover sites, speed and timing; (ii) that individuals utilise multiple wintering sites rather than single sites or even have a nomadic life-style; and (iii) that birds from a small breeding area in Sweden exhibit a wide wintering range throughout western sub-Saharan Africa. The departure dates from the wintering site differed between individuals (1-27 April), which may potentially be explained by differences in local environmental conditions, and was strongly correlated to arrival to the breeding grounds. The consistent high spring migration speed indicates a time-minimization strategy for the migratory journey and also highlights the importance of the timing of departure. In the future, repeated tracks of individual great reed warblers might offer fascinating insights how constrained or flexible long-distance migrating passerines are in their routes and timing of migration. 

## Supporting Information

Figure S1 **Inferred migration route and timing of male great reed warblers determined by geolocators.** Hexagons indicate breeding site (black), stop-over sites during autumn (orange) and spring (green) as well as wintering sites (blue). Lines with arrows indicate autumn migration routes (orange), winter movements (blue) and spring migration (green) whereby some parts are covered with slower migration speed (filled line indicate speed of <500 km/d) than other (double lines indicate speed of >500 km/d). Dashed lines represent great circle routes and do not necessarily represent the actual route taken by the bird due to analytical reasons (due to missing data; see text). Dates inferred from latitudinal and longitudinal data are given for departure and arrival at each site (in black); dates inferred from longitudinal movements only are given in grey. 1) The black bar in C (7V-50) indicates a stop-over where the actual position could not be determined. 2) In H (7V-72), only longitudinal data is given for the last position and the actual position is located anywhere along the grey line.
(PDF)

Table S1 **Sun angles for different stages during the year for each individual.** Breeding site before autumn migration (Sweden (departure)), autumn migration, wintering site after autumn migration until change to next wintering site (autumn equinox), second wintering site before spring migration (spring equinox), spring migration, and breeding site after spring migration (Sweden (arrival)) are given.
(XLSX)

Table S2 **Accuracy of geolocator position data at the breeding site based on distances between measured and known geographical positions.**
(XLSX)

Table S3 **Geolocator data over the annual cycle for individual great reed warblers.**
(XLSX)
