# Supplementary material for: Annual Cycle and Migration Strategies of a Trans-Saharan Migratory Songbird: A Geolocator Study in the Great Reed Warbler
Source: PLoS One. 2013 Oct 18;8(10):e79209. doi: 10.1371/journal.pone.0079209 (PMC3799637; doi:10.1371/journal.pone.0079209)
Supplement: Figure S1 — Inferred migration route and timing of male great reed warblers determined by geolocators. Hexagons indicate breeding site (black), stop-over sites during autumn (orange) and spring (green) as well as wintering sites (blue). Lines with arrows indicate autumn migration routes (orange), winter movements (blue) and spring migration (green) whereby some parts are covered with slower migration speed (filled line indicate speed of <500 km/d) than other (double lines indicate speed of >500 km/d). Dashed lines represent great circle routes and do not necessarily represent the actual route taken by the bird due to analytical reasons (due to missing data; see text). Dates inferred from latitudinal and longitudinal data are given for departure and arrival at each site (in black); dates inferred from longitudinal movements only are given in grey. 1) The black bar in C (7V-50) indicates a stop-over where the actual position could not be determined. 2) In H (7V-72), only longitudinal data is given for the last position and the actual position is located anywhere along the grey line. (PDF) [file pone.0079209.s001.pdf]

Figure S1 A-J. Inferred migration route and timing of male great reed warblers determined by geolocators. Hexagons indicate breeding site (black), stop-over sites during autumn (orange) and spring (green) as well as wintering sites (blue). Lines with arrows indicate autumn migration routes (orange), winter movements (blue) and spring migration (green) whereby some parts are covered with slower migration speed (filled line indicate speed of <500 km/d) than other (double lines indicate speed of >500 km/d). Dashed lines represent great circle routes and do not necessarily represent the actual route taken by the bird due to analytical reasons (due to missing data; see text). Dates inferred from latitudinal and longitudinal data are given for departure and arrival at each site (in black); dates inferred from longitudinal movements only are given in grey. 1) The black bar in C (7V-50) indicates a stop-over where the actual position could not be determined. 2) In H (7V-72), only longitudinal data is given for this position and the actual position is located anywhere along the grey line.

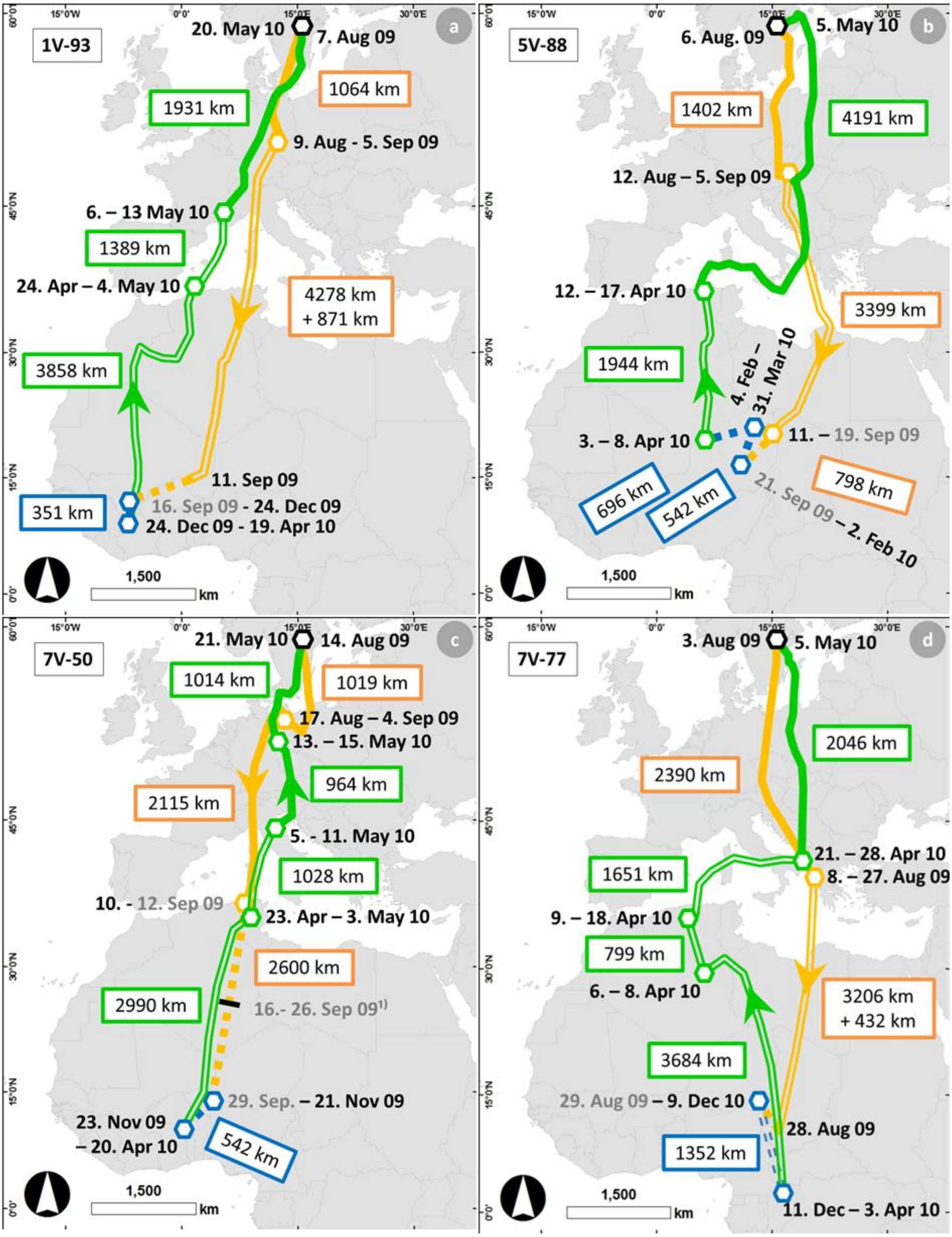
1V-93
20. May 10
7. Aug 09
1064 km
1931 km
9. Aug - 5. Sep 09
6. – 13 May 10
1389 km
24. Apr – 4. May 10
4278 km
+ 871 km
3858 km
11. Sep 09
351 km
16. Sep 09 - 24. Dec 09
24. Dec 09 - 19. Apr 10
1,500
km
5V-88
6. Aug. 09
5. May 10
1402 km
4191 km
12. Aug – 5. Sep 09
12. – 17. Apr 10
3399 km
1944 km
4. Feb –
31. Mar 10
3. – 8. Apr 10
11. – 19. Sep 09
696 km
542 km
798 km
21. Sep 09 – 2. Feb 10
1,500
km
7V-50
21. May 10
14. Aug 09
1014 km
1019 km
17. Aug – 4. Sep 09
13. – 15. May 10
964 km
2115 km
5. - 11. May 10
1028 km
10. - 12. Sep 09
23. Apr – 3. May 10
2600 km
2990 km
16.- 26. Sep 09 1)
29. Sep. – 21. Nov 09
23. Nov 09
– 20. Apr 10
542 km
1,500
km
7V-77
3. Aug 09
5. May 10
2046 km
2390 km
21. – 28. Apr 10
1651 km
8. – 27. Aug 09
9. – 18. Apr 10
799 km
6. – 8. Apr 10
3206 km
+ 432 km
3684 km
29. Aug 09 – 9. Dec 10
28. Aug 09
1352 km
11. Dec – 3. Apr 10
1,500
km

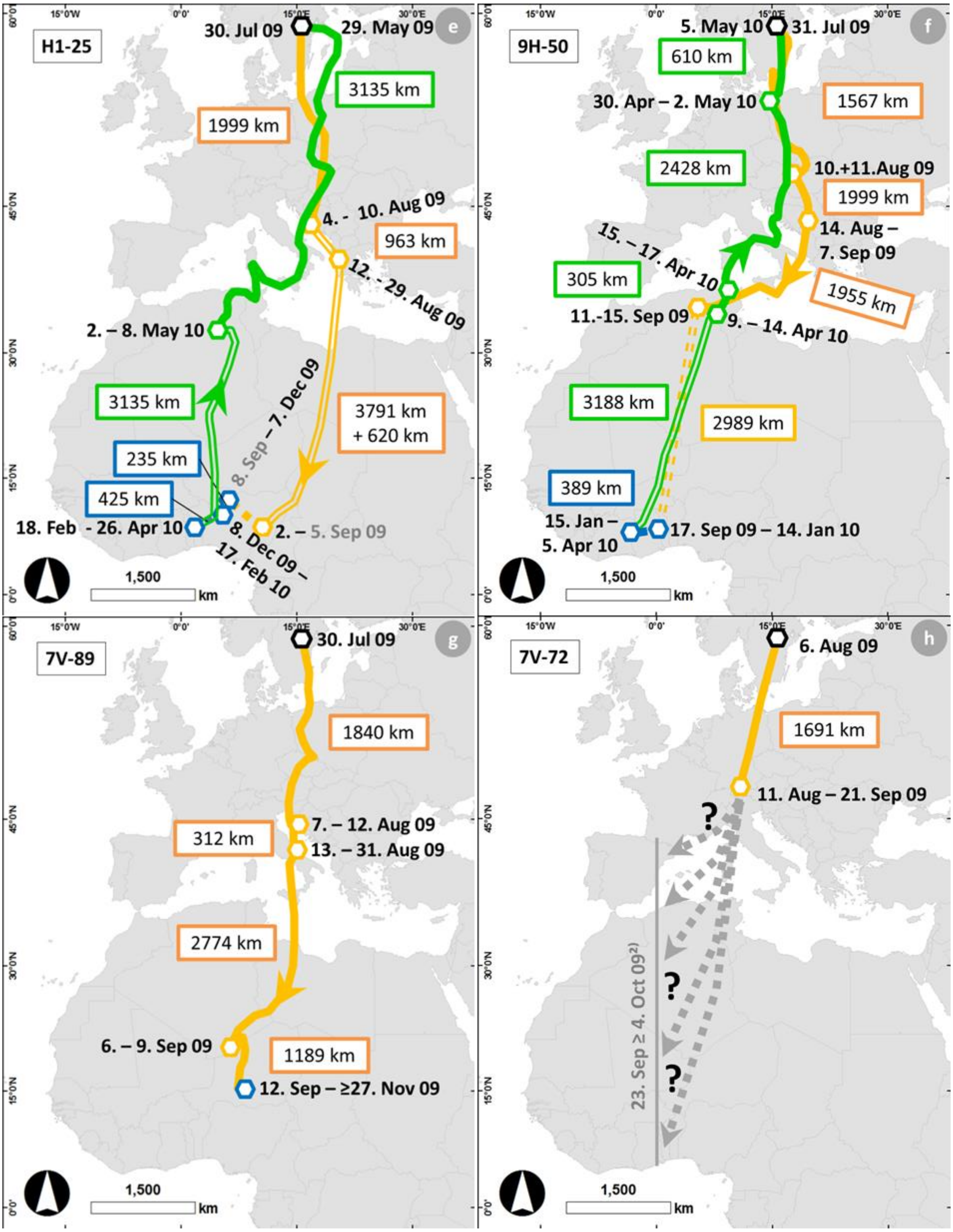
H1-25
e
30. Jul 09
29. May 09
3135 km
1999 km
4. - 10. Aug 09
963 km
12. - 29. Aug 09
2. – 8. May 10
8. Sep – 7. Dec 09
3791 km
+ 620 km
3135 km
235 km
425 km
18. Feb - 26. Apr 10
8. Dec 09 – 17. Feb 10
2. – 5. Sep 09
1,500
km
9H-50
f
5. May 10
31. Jul 09
610 km
30. Apr – 2. May 10
1567 km
2428 km
10.+11.Aug 09
1999 km
14. Aug – 7. Sep 09
15. – 17. Apr 10
305 km
1955 km
11.-15. Sep 09
9. – 14. Apr 10
3188 km
2989 km
389 km
15. Jan – 5. Apr 10
17. Sep 09 – 14. Jan 10
1,500
km
7V-89
g
30. Jul 09
1840 km
7. – 12. Aug 09
312 km
13. – 31. Aug 09
2774 km
6. – 9. Sep 09
1189 km
12. Sep – ≥27. Nov 09
1,500
km
7V-72
h
6. Aug 09
1691 km
11. Aug – 21. Sep 09
?
?
?
23. Sep ≥ 4. Oct 09²⁾
1,500
km

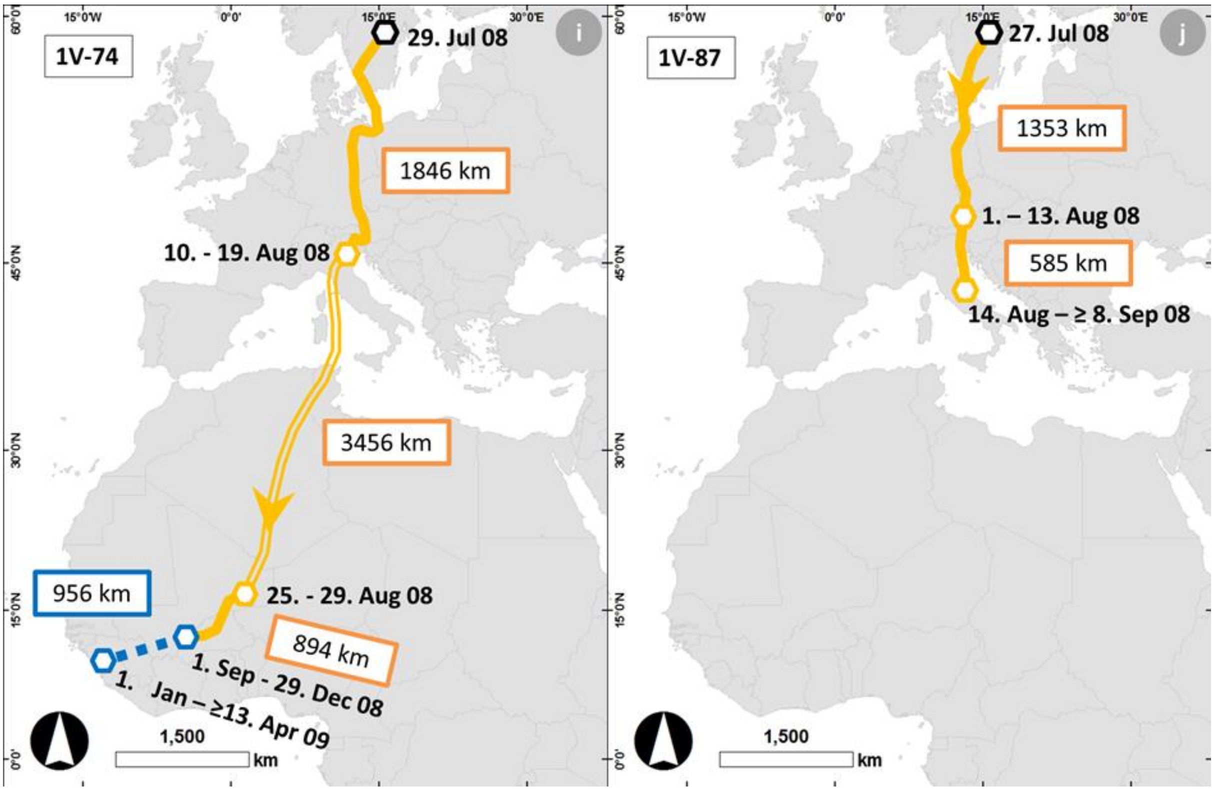
1V-74
i
29. Jul 08
1846 km
10. - 19. Aug 08
3456 km
25. - 29. Aug 08
956 km
894 km
1. Sep - 29. Dec 08
1. Jan – ≥13. Apr 09
1,500
km
1V-87
j
27. Jul 08
1353 km
1. – 13. Aug 08
585 km
14. Aug – ≥ 8. Sep 08
1,500
km
